# Immunobiology roles of the human CD161 receptor in T cells

**DOI:** 10.3389/fimmu.2025.1648305

**Published:** 2025-08-14

**Authors:** Bainan Tong, Mingxuan Wang, Lu Liu, Xinyue Yang

**Affiliations:** Department of Ophthalmology, The Second Hospital of Jilin University, Changchun, China

**Keywords:** CD161, LLT1, T cells, Th17 cells, MAIT cells

## Abstract

Human C-type lectin-like molecule CD161 is a type II transmembrane protein expressed on the surface of various lymphocytes within both the innate and adaptive immune systems. CD161 serves as a marker for innate-like T cells and IL-17-producing cells. However, the meaning of these T cells expressing CD161 has not yet been fully determined. Is CD161 merely a phenotypic marker used to identify T cells that are in a common state of activation and/or lineage? Or does this C-type lectin itself have important effector functions? This article aims to explore the latest research progress on CD161-expressing T cells, particularly human αβTCR+T and γδTCR +T cells, and evaluate the importance of CD161 expression on immune function and human diseases through this research.

## 1 Introduction

**Figure 1:**
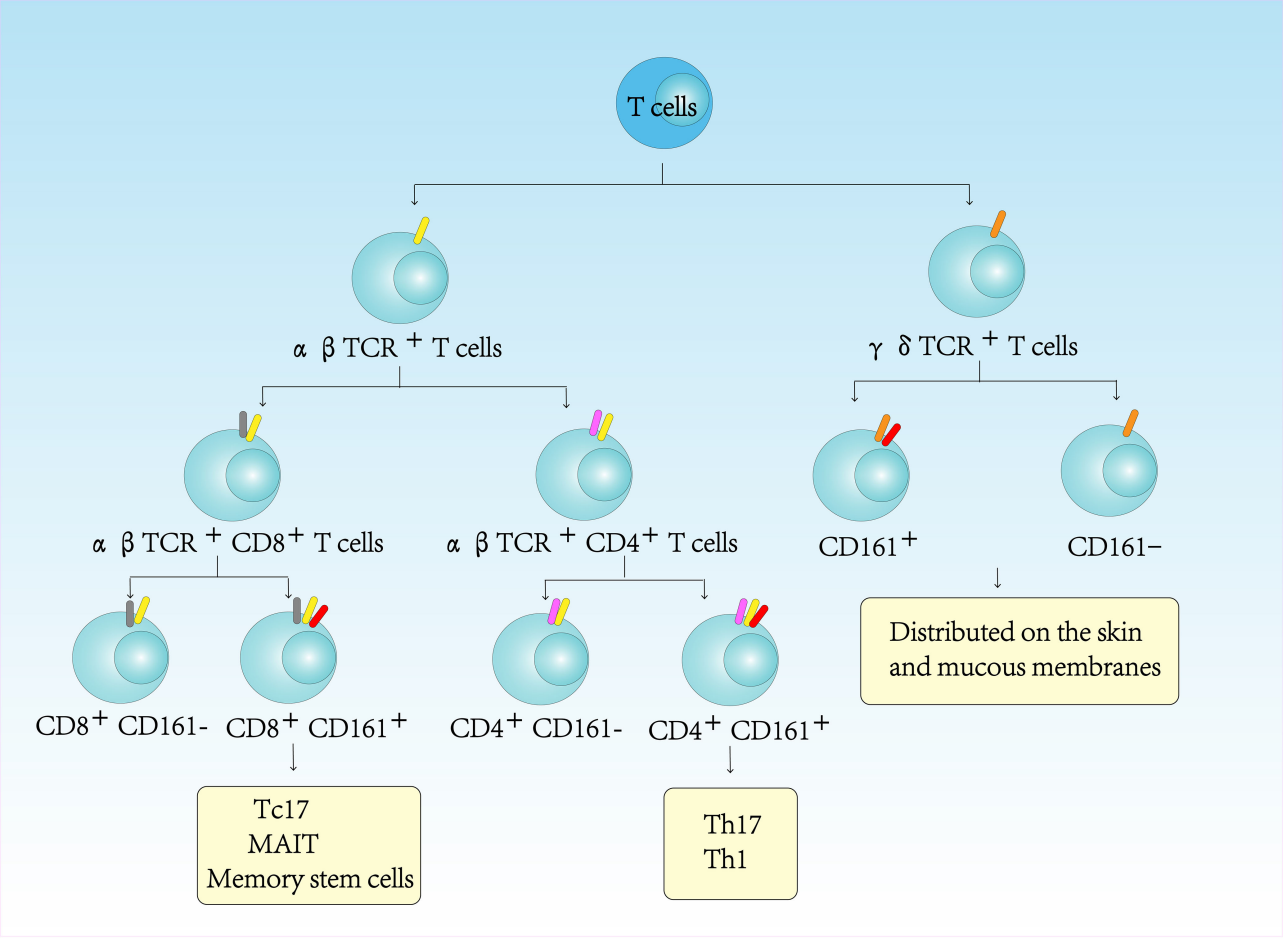
CD161-expressing in human T cells immune-phenotyping.

CD161, a type II transmembrane protein of the C-type lectin family encoded by KLRB1 within the NK cell gene complex (chromosome 12), shares 46-47% homology with rodent NKRP1 glycoprotein (1). CD161 is a 40kDa dimer composed of two subunits, which is expressed in most human NK cells and approximately 24% of peripheral blood T and intestinal T cells. Only a small fraction of CD161 is expressed in NKT cells, which account for only 1% of all T cells in lymphoid tissues and liver (2, 3). In peripheral blood lymphocytes, CD161 is not only expressed in CD4+TCRαβ+T cells, but also in CD8+TCRαβ+T cells, CD4-CD8-TCRαβ+T cells, and CD4-CD8- TCRγδ+T cells (4–6) (**Figure 1**). The lectin-like transcript 1 (LLT1) has been identified as a ligand for CD161. LLT1 is not constitutively expressed in quiescent cells, but its expression is upregulated upon cellular activation. It can be detected on the surface of activated B cells, T cells, NK cells, monocytes, and dendritic cells (7). The interaction between CD161 and LLT1 exhibits low affinity and rapid kinetics, hallmark features of intercellular recognition receptors (7). It is believed that the CD161-LLT1 receptor-ligand pairs play a role in facilitating communication between different CD161-expressing cells and LLT1-expressing cells within the innate and adaptive immune system (8).

## 2 CD161 ligands

Lectin-like transcript 1 (LLT1), a type C lectin-like protein, was initially characterized by Boles et al. It has been identified as the ligand for CD161 receptor, revealing an immunoregulatory lectin-ligand pair (9). C-type lectin-like proteins are capable of binding to a wide range of ligands, thereby eliciting a diverse array of responses. These responses encompass various biological processes, including endocytosis, pro-inflammatory signaling, and anti-inflammatory signaling (10).

LLT1 mediates CD161-dependent enhancement of TCR-induced IFN-γ production, suggesting its immunomodulatory role. Interestingly, the expression of CD161 on T cells and NKT cells is more frequent than that on other NK receptors. It is necessary to assess its correlation with the disease (9). In rheumatoid arthritis synovial fluid, LLT1 expression is significantly upregulated in monocyte subsets exhibiting more differentiated and mature phenotypes. Within the synovial tissue of rheumatoid arthritis patients, LLT1-expressing cells were identified in the synovial layer, subsynovial layer, and lymphocyte-infiltrated regions. The staining pattern of LLT1 closely resembled that of CD68. Flow cytometry analysis conducted on digested synovial tissue further confirmed that LLT1 was specifically expressed on CD68-positive cells (11).

When investigating the LLT1/CD161 interaction and its consequences, the precise characteristics of the antibodies employed remain ambiguous (e.g., agonistic effects, antagonistic effects, or redirected activation achieved via antibody-dependent cell-mediated cytotoxicity). This ambiguity may elucidate the underlying mechanisms responsible for the diverse effects observed when these antibodies are utilized in varying contexts (10). Overall, it is precisely the induction of this inhibitory signal that has prompted some researchers to propose that LLT1 shares similarities with MHC Class I molecules and contributes to the “missing self” hypothesis (12). Consequently, in the context of infectious diseases and cancers, elucidating the expression and function of LLT1 and CD161 holds significant importance. In the Cancer Genome Atlas (TCGA), CD161 exhibits significantly differential expression across most tumor types and is associated with improved survival outcomes (13). Studies have elucidated the prognostic significance of LLT1 expression in both tumor and stromal components of oral squamous cell carcinoma (OSCC), along with its potential utility in enhancing prognostic prediction and patient stratification (14). Additionally, the expression of CD161 demonstrates a significant correlation with the immunomodulatory interactions between lymphocytes and non-lymphocytic cells. Notably, CD161 expression is closely linked to T cell infiltration, immune checkpoint regulation, immune-activating genes, immunosuppressive genes, chemokines, and chemokine receptors (13, 15).

The interaction between CD161 and its ligand, LLT1, has been documented to transmit inhibitory signals. For instance, it can suppress the functions of NK cells. In glioma, the CD161 - CLEC2D signaling pathway has been shown to attenuate the cytotoxicity of CD8+ T cells (16). Conversely, within T cells, CD161 demonstrates an activating role. Specifically, the TL1A - DR3 signaling axis enhances the production of IFN-γ via CD161 (17). We postulate that CD161 may exhibit “signal bipolarity”. Its functional outcome is contingent upon the co - receptor context. When co - expressed with PD-1, CD161 exerts inhibitory effects (18). In contrast, when co - expressed with DR3 or IL-23R, it assumes an activating role (19).

## 3 CD161 expression in αβTCR ^+^ CD4^+^T cells

CD161 has been widely recognized as a surface marker of IL-17-producing T cells, particularly Th17 lymphocytes (20). Substantial evidence demonstrates that circulating and gut-resident CD161+CD4+ T cells exhibit characteristic Th17 features and serve as the primary source of IL-17 upon stimulation, unlike their CD161- counterparts (21).

In Crohn’s disease, CD161+ cells exhibit an activated Th17 phenotype characterized by elevated expression of IL-17, IL-22, and IL-23 receptors. Unlike healthy controls requiring IL-1β co-stimulation, Crohn’s disease-derived CD161+CD4+ T cells respond to IL-23 alone by producing IL-17 and IFN-γ (22). Circulating CD161+Th17 cells are labeled for intestinal homing, as illustrated by high levels of CC chemokine receptor 6 and integrin β7 expression. CD161+Th17 cells support their colonic phenotype by increasing the number of inflammatory infiltrates in Crohn’s disease lesions and the number of inflammatory mediators induced by intestinal cells (22). The CD161+CD39+ co-expression pattern appears particularly effective for identifying Th17-enriched populations, with these cells displaying characteristic Th17 markers (CCR6, IL-23R) and serving as useful indicators of Th17 activity in Crohn’s disease (23). Similar pathogenic involvement occurs in juvenile idiopathic arthritis, where CD4+CD161+ T cells producing IL-17A and IFN-γ correlate with disease activity markers (24).

The pathogenic role of CD161+ T cells extends to other autoimmune conditions. In giant cell arteritis and polymyalgia rheumatica, CD161-expressing Th1 and Th17 lymphocytes show distinct expansion patterns compared to healthy controls; reduced Treg and Th1 populations accompany significantly increased Th17 frequencies (25). Temporal artery biopsies reveal substantial Th17/Th1 infiltration in affected patients, with CD161+CD4+ T cells - considered Th17 precursors - demonstrating enhanced IL-17 production capacity linked to decreased STAT1 phosphorylation (25). Rheumatoid arthritis similarly demonstrates disease activity-associated fluctuations in circulating CD161+Th17 and CD161+Th1 cell percentages, supporting the Th17 involvement hypothesis and suggesting CD161+ subset imbalances contribute to RA development (26).

Beyond autoimmunity, in other diseases such as chronic hepatitis B virus (HBV) infection, CD161+CD4+ T cells are involved in mediating antiviral, pro-inflammatory, and pro-fibrotic responses. Compared with their homologous CD161-CD4+ T cell counterparts, CD161+CD4+ T cells exhibited enhanced production of pro-inflammatory cytokines, such as interleukin (IL)-17 and interferon (IFN)-γ, and demonstrated elevated expression levels of liver-homing chemokine receptors, including CCR6, CXCR6, and CX3CR1 (27). Evidence indicates that in spontaneously hypertensive rats (SHRs), the capacity for pro-inflammatory and pro-hypertensive IL-17F expression driven by RORγt is significantly augmented upon activation of innate immunity. The elevated levels of RORγt and IL-17F contribute to the development of hypertension in SHRs and may represent potential therapeutic targets (28).

While CD161 represents a valuable biomarker for IL-17-producing T cells (especially Th17 subsets), its standalone use for functional classification requires caution due to phenotypic heterogeneity among CD161-expressing populations.

## 4 CD161 expression in αβTCR ^+^ CD8^+^T cells

CD161high expression distinguishes two functionally distinct CD8+ T cell subsets: Tc17 cells and mucosal-associated invariant T (MAIT) cells, both thought to derive from a common CD161high progenitor. MAIT cells, which represent approximately 5% of human T cells, exhibit potent effector functions and preferentially localize to mucosal tissues. Notably, CD8+CD161^high^ T cells constitute up to 95% of peripheral blood CD8+ T cells in adults (29). MALT cells express T cell receptor Va7.2-Ja33, which is limited by non-classical Ib molecule MHCI and is enriched in mucosal sites (30).

In primary progressive multiple sclerosis (PP-MS), flow cytometry analyses reveal selective depletion of circulating CD8+CD161^high^ T cells (primarily MAIT cells), while CD8+CD161^int^ populations remain intact (31). Conversely, chronic hepatitis B infection is associated with expansion of non-MAIT CD161+CD8+ T cells, which display reduced antiviral capacity but enhanced pathogenic potential. This supports the idea of CD161 expression as a marker of pathogenic CD8+T cell subsets and provides an intervention target for liver injury (32).

Beyond their role in antimicrobial defense, CD8+CD161^high^ T cells participate in sterile inflammation. These cells accumulate in the livers of patients with chronic hepatitis C, autoimmune hepatitis, and non-alcoholic fatty liver disease (33). CD8+CD161^high^ T cells have been found in the brains of multiple sclerosis patients and are believed to be pathogenic (34). During SIV infection, CD161+CD8+ T cells maintain Th1-like cytotoxicity while increasing IL-17 production—a potential compensatory mechanism for intestinal Th17 cell loss (35).

The CD8+CD161^high^ population exhibits unique functional properties, including:(1) Polyfunctional effector capacity: High expression of cytotoxic mediators (granzymes, perforin) and transcription factors (T-bet, EOMES). CD161+CD8+ T cells of SIV-infected macaque monkeys show stronger IL-17 production and maintain Th1- type and cytotoxic functions, while CD161+CD4+ T cells are inhibited in IL-17 and granzyme B production (35). JR Fergusson et al. showed that CD8+CD161^high^ T cells are characterized by enhanced versatility, elevated levels of cytotoxic mediators, and high expression of transcription factors T-bet and eomesodermin (EOMES) (36). (2) Tissue homing specialization: Enhanced adhesion molecule expression and blood-brain barrier penetrance. The research by Bryan Nicol et al. indicates that CD8^+^CD161 moderately expressed T cells exhibit effector cell characteristics, upregulate the expression of cell adhesion molecules, demonstrate enhanced capacity to cross the blood-brain barrier (32). (3) Cytokine versatility: Production of IL-17, IFN-γ, GM-CSF, and IL-22. The CD8+CD161^high^ T cell population in healthy individuals secretes IL-17 and exhibits a Th17-like differentiation profile, characterized by shared expression of RORγt transcription factor, cytokines (IL-17A, IL-22), chemokine receptors (CCR6, CXCR6, CCR2), and IL-23 receptor (37).

**Figure 2:**
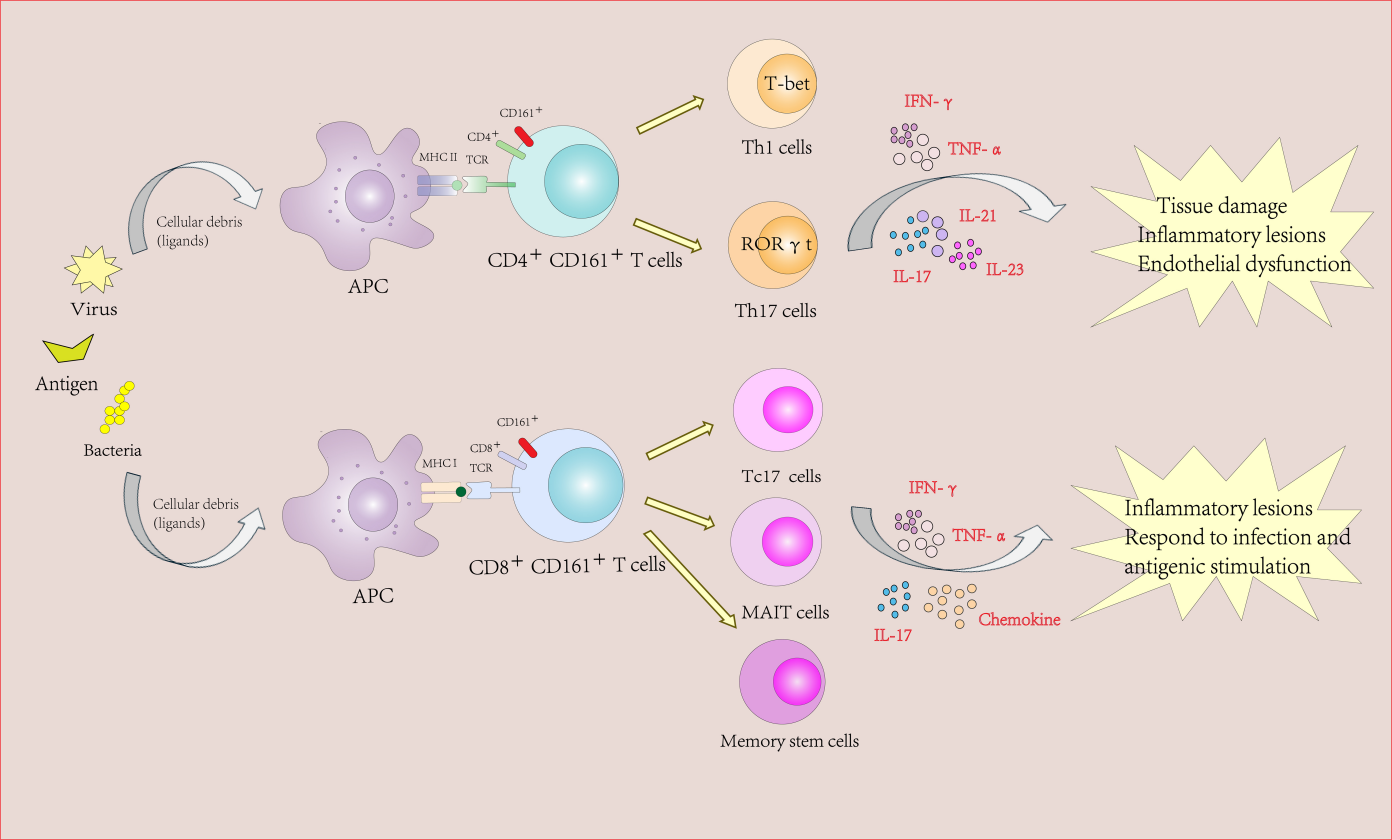
The role of CD161-expressing CD4+ and CD8+ Tcells in autoimmune diseases and inflammatory diseases.

Notably, adenovirus-based vaccines induce robust CD161^int^CD8+ T cell responses, highlighting their relevance for therapeutic vaccination strategies (31). The shared transcriptional program between CD161^high^CD8+ T cells and Th17 cells (RORγt, IL-23R, CCR6) further underscores their role in tissue-specific immunity (33) (**Figure 2**).

## 5 CD161 expression in γδTCR^+^T cells

CD161 identifies functionally distinct subsets across CD4+, CD8+, and γδTCR+ T cell populations that share the capacity for TCR-independent IFN-γ production upon IL-12/IL-18 stimulation (38, 39). This innate-like response pattern, observed particularly in CD161++ cells, occurs independently of CD161 receptor function itself (38).To date, only a few studies have reported that CD161+ T cells, especially CD161+CD4+ T cells, possess innate-like immune characteristics. For instance, when stimulated with IL-12 + IL-18, CD161+ Tconv and CD161+ Treg secrete significantly more IFN-γ than their CD161- counterparts, demonstrating innate-like immune properties. However, the production of cytokines is limited to CD161++ T cells. Unlike CD8+ T cells, these cells constitute only relatively small subpopulations in CD4+ T cells and TCRγδ+ T cells. CD161+CD4+ T cells expressing the promyelocytic leukemia zinc finger (PLZF) transcription factor isolated from the fetal small intestine can produce IFN-γ when stimulated by IL-12 + IL-18 (39). CD161+CD4+ T cells secrete interferon-γ (IFN-γ) upon stimulation with interleukin-12 (IL-12) and interleukin-18 (IL-18). This secretion can be significantly augmented by the engagement of death receptor 3 (DR3) with anti-DR3 monoclonal antibodies (mAbs) (19). Tumor necrosis factor-like cytokine 1A (TL1A), a ligand for DR3, augments the production of multiple pro-inflammatory cytokines by CD161+CD4+ T cells upon stimulation with IL-12 and IL-18 (17). Current evidence reveals that the expression level of cluster of differentiation 161 (CD161) is significantly elevated in fibrotic biopsy samples. CD161 is a marker of human memory T cells with innate immune-like functional potential and the ability to generate interleukin (IL)-17A. Compared with non-fibrotic renal tissues, the expression level of CD161 on TCRγδ+ T cells in fibrotic tissues is significantly increased (40).

However, the cytokine requirements for the TCR-independent activation of CD161+CD4+ T cells remain incompletely elucidated. Overall, while some studies propose that CD161 may serve as a marker for innate-like T cells, it remains uncertain whether CD161 expression alone is sufficient to reliably identify this specific T cell subset.

## 6 CD161 and memory phenotype

CD161 has been extensively studied in MR1-restricted T cell subsets, including IL-17-producing CD4+ T cells (TH17 MAIT cells) and CD8+ T cells (Tc17 cells). Non-MAIT, non-MR1-dependent CD161-expressing T cells also exist and are typically characterized as effector memory cells with a stem cell-like phenotype (41). Stimulation of interleukin-18 (IL-18) and interleukin-12 (IL-12) is known to induce interferon-γ (IFN-γ) production by natural killer cells (NK cells) and, to some extent, by T cells, but to a limited extent. The results show that CD161++ CD8+ T cell population is the main T cell population triggered by this mechanism. Both CD161++Vα7.2+ and CD161++Vα7.2− T cell subpopulations responded to IL-12+IL-18 stimulation, suggesting that this response was not limited to MAIT cells, but to the CD161++ phenotype. Bacteria and TLR agonists also indirectly trigger IFN-γ expression via IL-12 and IL-18. These data indicate that CD161++ T cells are the dominant T cell population that responds directly to IL-12+IL-18 stimulation (29, 42). Hester Koppejan et al. reported that in untreated early rheumatoid arthritis (RA) patients, mucosa-associated invariant T (MAIT) cells are predominantly CD4+ and exhibit reduced CD161 expression levels. These cells demonstrate lower reactivity upon stimulation compared to patients with spondyloarthritis (SpA) and healthy controls. Using CD161 as a marker for MAIT cells may lead to an underestimation of functionally impaired MAIT cells (43).

## 7 CD161+T cells in autoimmune disease

CD161+ T cells play significant roles in multiple autoimmune disorders, demonstrating distinct pathological mechanisms across different disease contexts. In IgG4-related disease (IgG4-RD), these cells contribute to immune dysregulation through an altered Treg/Teff balance, with CD161+ Tregs potentially driving disease pathogenesis (44).

Rheumatoid arthritis (RA) exhibits particularly strong associations with CD161+ T cell activity. In rheumatoid arthritis synovial fluid (RASF) analysis reveals: (1) elevated frequencies of CD4+CD161+ T cells alongside increased CD147 and CD98 expression (43); (2) accumulation of CD161+CD39+ and CD39+CD73+ microparticles that correlate with disease characteristics and modulate chemokine (CCL17/20/22) and cytokine (IL-17/10) production in synoviocytes and PBMCs (44); and (3) an inverse relationship between CD4+CD161+ and CD4-CD8-CD161+ T cell populations that correlates with disease activity, suggesting CD4+CD161+ cells promote local joint inflammation (45) Notably, methotrexate treatment alters the Th17-derived Th1 to CD161+Th17 cell ratio in early-onset RA, suggesting therapeutic modulation of these populations (45).

Primary Sjögren’s syndrome (pSS) presents a more complex scenario, where both RORγt+ (Th17) and RORγt- CD161+ CD4+ T cell subsets demonstrate pathogenic potential. While Th17-like cells contribute to humoral manifestations, the RORγt- subset shows stronger association with overall disease activity (46). Patients with pSS exhibit increased IL-17 production from CD161+ T cell subsets, with elevated frequencies of CD4+CD161+ T cells and their effector populations showing positive correlation with both disease activity indices and clinical severity, suggesting their pathogenic involvement and potential as therapeutic targets in pSS (47).

The developmental biology of these pathogenic subsets traces back to CD161+ precursors in cord blood and thymus, with mature Th17 cells maintaining characteristic features including RORγt expression and production of IL-17A/F/22. These findings collectively position CD161+ T cells as both biomarkers and potential therapeutic targets in autoimmune pathogenesis, though their exact roles vary significantly across different disease microenvironments (48).

## 8 CD161+T cells in cancer

CD161 has been proposed as a potential cancer biomarker. Functional enrichment analysis revealed that CD161 and its co-expressed genes were significantly associated with multiple cancer-related and immune signaling pathways, indicating their potential involvement in the immune response during carcinogenesis. Additionally, immune infiltration analysis demonstrated that CD161 expression was correlated with immune cell infiltration (13, 49, 50). CD161 demonstrates dual potential as both a prognostic biomarker for breast cancer outcomes and a therapeutic target for immunotherapy, where its cooperative regulation of the immune microenvironment with other checkpoints may inform novel combination treatment strategies (12, 52). T cells showed higher LLT1 levels than tumor-infiltrating lymphocytes (TILs), while CD161 was highly expressed in CD8+T cells at the tumor front but decreased in paracancer tissue. When the expression level of TC-derived LLT1 is high, the clinical prognosis of patients is poor. TILs of CD161+ and LLT1+ were associated with better prognosis (51). Experimental evidence has established that CD161-CLEC2D interaction mediates suppression of natural killer (NK) cell cytotoxic activity. Immunohistochemical analyses have verified CLEC2D expression in glioma cells, demonstrating functional activation of this inhibitory axis in glioblastoma pathogenesis. By using CRISPR-Cas9 technology to edit the KLRB1 gene in human T cell co-culture experiments and humanized glioblastoma mouse models to inhibit this pathway, not only was T cell-mediated cytotoxicity enhanced, but tumor growth was also slowed and survival was prolonged (16). A meta-analysis revealed that the expression of CLEC2D (encoding LLT1) and KLRB1 (encoding CD161) genes was positively correlated with favorable prognosis in non-small cell lung cancer (NSCLC), independent of the extent of T-cell and B-cell infiltration. These findings are consistent with the positive influence of LLT1/CD161 on NSCLC patient survival and suggest that CD4+ T cells expressing CD161 represent promising candidates for mediating an effective anti-tumor recall response (52).

CD4+CD161+ Tem cells demonstrate enhanced TCR responsiveness under suboptimal cytokine conditions, mediated through SOX4 and cytokine receptor upregulation. Their therapeutic potential is underscored by vaccine-induced expansion and TGFβ1-mediated suppression, suggesting combination strategies with TGFβ inhibition (53). Further complexity emerges in hepatocellular carcinoma, where CD8+PD-1+CD161+ and CD8+PD-1+CD161− T cells display distinct functional and spatial distributions within tumors (54) (**Table 1**).

**Table 1:** Abstract of the experimental findings regarding the structure and function of CD161 in the published literature.

Category	Key Findings	Implications	References
Molecular Characteristics	- Type II transmembrane protein of C-type lectin family encoded by KLRB1 (chromosome 12) - 40kDa dimer with 46-47% homology to rodent NKRP1 - Ligand: Lectin-like transcript 1 (LLT1/CLEC2D), binding exhibits low affinity/rapid kinetics - Expressed on NK cells (most), T cells (~24% peripheral blood/intestinal), rare in NKT cells (<1%)	- Evolutionary conservation suggests critical immune function - Broad expression pattern indicates pleiotropic roles	(1, 2, 7, 9)
Expression Patterns	- CD4+ subsets: Th17, Treg, innate-like T cells - CD8+ subsets: MAIT cells (Va7.2+MR1-restricted), Tc17, tissue-resident memory - γδT cells: Innate-like responders - Cancer: Heterogeneous (high in tumor-front CD8+ T cells VS. low in stroma)	- Subset-specific expression correlates with functional specialization - Microenvironment-dependent regulation	(4, 21, 29, 37, 51)
Functional Mechanisms	Inhibitory: - CD161-CLEC2D suppresses NK/CD8+ T cell cytotoxicity (glioma) - Co-expression with PD-1 enhances immune exhaustion Activating: - TLIA-DR3-CD161 axis boosts IFN-y production - IL-12/IL-18 induces TCR-independent IFN-y (innate-like response) - IL-23R co-expression drives Th17 polarization	- "Signal bipolarity" context-dependent on: • Ligand density (LLT1) • Co-receptors (PD-1 VS. DR3) • Cytokine milieu (IL-12/IL-23) - Therapeutic targetability via pathway modulation	(12, 16, 17, 19, 34)

## 9 Conclusion

CD161, as a C-type lectin-like receptor, has shown significant progress in research regarding its expression patterns and functional roles in T cells, but there are still several key issues that remain unsolved. The limitations of CD161 as a marker for Th17 cells. Although CD161 is widely regarded as a surface marker of Th17 cells (21), its specificity is highly controversial: 1. False positive issue: Only about 60% of CD161^high^CD4+ T cells actually secrete IL-17 (24), suggesting that CD161 may mark a broader population of activated T cells rather than a specific Th17 subset. 2. Functional heterogeneity: In Crohn’s disease, CD161+ T cells simultaneously produce IL-17 and IFN-γ (22), this “Th1/Th17 mixed phenotype” challenges the paradigm of CD161 as a pure Th17 marker. Therefore, we propose a new perspective that CD161 may more accurately identify a “plastic precursor cell”, whose differentiation direction (Th1/Th17) depends on the local cytokine environment (such as IL-12 vs. IL-23), rather than the terminal effector phenotype.

Re-evaluating the Relationship Between CD161 and MAIT Cells. The literature generally equates CD161^high^CD8+ T cells with MAIT cells (29), but this association has the following issues: 1. Technical bias: Most studies rely on co-labeling of CD161 and Vα7.2, but the CD161^high^Vα7.2− population also has IL-12/IL-18 responsiveness (42), suggesting that CD161 may function independently of the MR1 signaling pathway. 2. Functional conflict: The classic definition of MAIT cells is “bacterial reactivity”, but CD161^high^CD8+ T cells are also enriched in non-infectious inflammation, suggesting that their function may extend beyond antibacterial immunity. Therefore, we believe that CD161 may represent a universal marker of “innate-like T cells”, whose function is not limited to MAIT cells, but rather rapidly initiates inflammatory responses through low-threshold responses to cytokines (such as IL-12/IL-18).

CD161 is by no means a simple phenotypic marker. Instead, its functions exhibit a high degree of environmental dependence, assuming multifaceted roles in Th17 differentiation, innate - like responses, and immune regulation. The central conflict in current research lies in the over - simplification of its biological significance. Specifically, CD161 may inherently function as an “immune response regulator” rather than a marker exclusive to a particular subset. In the future, it is necessary to delve into its signaling pathways and integrate multi - dimensional data to construct a more accurate functional atlas of CD161, thereby providing guidance for clinical translation.
